# Room temperature green synthesis and time resolved kinetics of formation of xanthan gum stabilised silver nanoparticles with catalytic and antibacterial potential

**DOI:** 10.1038/s41598-025-17416-0

**Published:** 2025-08-27

**Authors:** George Mallouka, Omar Karkoutly, Omar Karzoun, Fatima AlHannan, Sultan Akhtar, Fryad Henari, G. Roshan Deen

**Affiliations:** 1 Materials for Medicine Research Group, School of Medicine, Royal College of Surgeons, Medical University of Bahrain, Busaiteen, 228 Kingdom of Bahrain; 2 Department of Biophysics, Institute for Research and Medical Consultations, Imam Abdulrahman Bin Faisal University, Dammam, 31441 Kingdom of Saudi Arabia

**Keywords:** Green chemistry, Polymer chemistry

## Abstract

Well-defined and stable, spherical silver nanoparticles (AgNPs) were synthesized at room temperature by a green approach using xanthan gum (XG) as a reducing and stabilizing agent. The effect of various concentrations of silver nitrate on the formation kinetics of AgNPs at 23 °C and pH 10 was studied. The nanoparticles formation with XG as a reducing agent followed a first-order reaction kinetics. The denaturation of XG from a helix to a random polymer coil was achieved at 23 °C and pH 10. Renaturation of XG was not observed at room temperature, and the nanoparticles were stable against aggregation during prolonged storage of more than 8 months. The formation of AgNPs was studied using UV-Vis absorption spectroscopy, and a strong surface plasmon resonance peak centred around 407–413 nm confirmed the presence of nanoparticles. The optical bandgap of the nanoparticles was estimated to be in the range of 2.46 to 2.55 eV. Transmission electron microscope (TEM) images showed spherical and non-agglomerated nanoparticles of 10–22 nm size range. The Fourier-transform infra-red spectroscopy (FTIR) revealed the presence of an organic layer (due to XG) on the surface of the nanoparticles. The nanoparticles were highly effective in the degradation of a model organic pollutant, 2-nitrophenol, exhibiting 80% degradation within 10 min, with a pseudo-first-order rate constant of 0.211 min^− 1^. The AgNPs showed size-dependent favourable antibacterial potential against *Staphylococcus aureus* and *Salmonella typhimurium*.

## Introduction

Nanotechnology has been widely exploited in the synthesis of various organic, inorganic and hybrid materials for targeted biomedical and engineering applications^1–3^. Physical, chemical and biological methods have been used to develop nanomaterials with tuneable morphological parameters. However, both physical and chemical methods are not environmentally friendly, require sophisticated laboratory equipment and involve various (toxic) organic solvents. In order to overcome such issues, green approaches using biological materials, especially the aqueous extracts of various plants and plant products, have been employed as an alternative method. Plant extracts are rich in phytochemicals such as sugar, terpenoids, polyphenols, alkaloids, phenolic acids, proteins, flavonoids etc. and these are involved in redox reactions that reduce the metal ions to metal nanoparticles^4–8^.

The synthesis of AgNPs by green approaches has attracted extensive attention over the past decade due to its wide range of applications in chemical sensing, cancer diagnosis and therapy, antimicrobial coatings, and catalysis^9–11^. Other than the extracts of plants, natural biopolymers such as lentinan^12^ agar^13^ starch^14^ locust bean gum^15^ gellan gum^16^ gum Arabic^17^ and XG^18,19^ have also been used in the green synthesis of AgNPs. These biopolymers are a rich source of polysaccharides and are biocompatible. Polysaccharides contain many hydroxyl groups (−OH) which acts both as a mild reducing and stabilizing or capping agent. The well-defined structure of the biopolymers stabilises the nanoparticles against aggregation through non-covalent interactions. The biopolymers can further undergo conformational changes in response to external temperature changes which influence the structure and stability of the nanoparticles.

XG is a type of extracellular polysaccharide which is secreted by the bacterium *Xanthomonas campestris* by fermentation^20^. The gum is resistant to enzymatic degradation and is stable in a wide range of temperatures and pH. The structure consists of a linear D-glucose backbone linked alternately to a trisaccharide side chain. The side chain consists of an acetyl group with D-mannose, D-glucuronic acid, and a terminal D-mannose unit linked to a pyruvate group. The ratio of D-glucose, D-mannose and D-glucuronic acid is in the ratio of 2:2:1. The morphological, structural, physico-chemical and rheological properties of XG have been studied. XG-capped silver and palladium nanoparticles have been demonstrated to have antibacterial and catalytic properties^18,19^. XG-based hybrid hydrogels containing AgNPs have emerged as a promising platform for accelerated tissue generation in skin defects^21^. Due to its non-toxicity and biocompatibility, XG has extensive applications in the pharmaceutical and food industries^22^. XG exists as double-stranded helix which can undergo a reversible uncoiling to a single disordered chain in water at temperatures higher than 60 °C. This uncoiling exposes the many -OH groups present in the polymer which act as mild reducing agents. This has been exploited in the synthesis of metallic nanoparticles using XG.

In line with our research interest in green nanotechnology and the structure-property-application relationship of new nano/functional materials, we report the XG-mediated green synthesis of well-defined and stable AgNPs at room temperature (23 °C) and their time-resolved kinetics of formation. In this study, the chain conformation of XG from helix to random coil at room temperature was achieved in a basic pH medium to enhance the reduction and stabilization process. The antibacterial and catalytic properties of XG-capped AgNPs were also evaluated. This study is expected to enrich the information on the synthesis of polysaccharide-conjugated well-defined AgNPs for possible biomedical applications.

## Results and discussion

### XG-mediated green synthesis of AgNPs

**Fig. 1:**
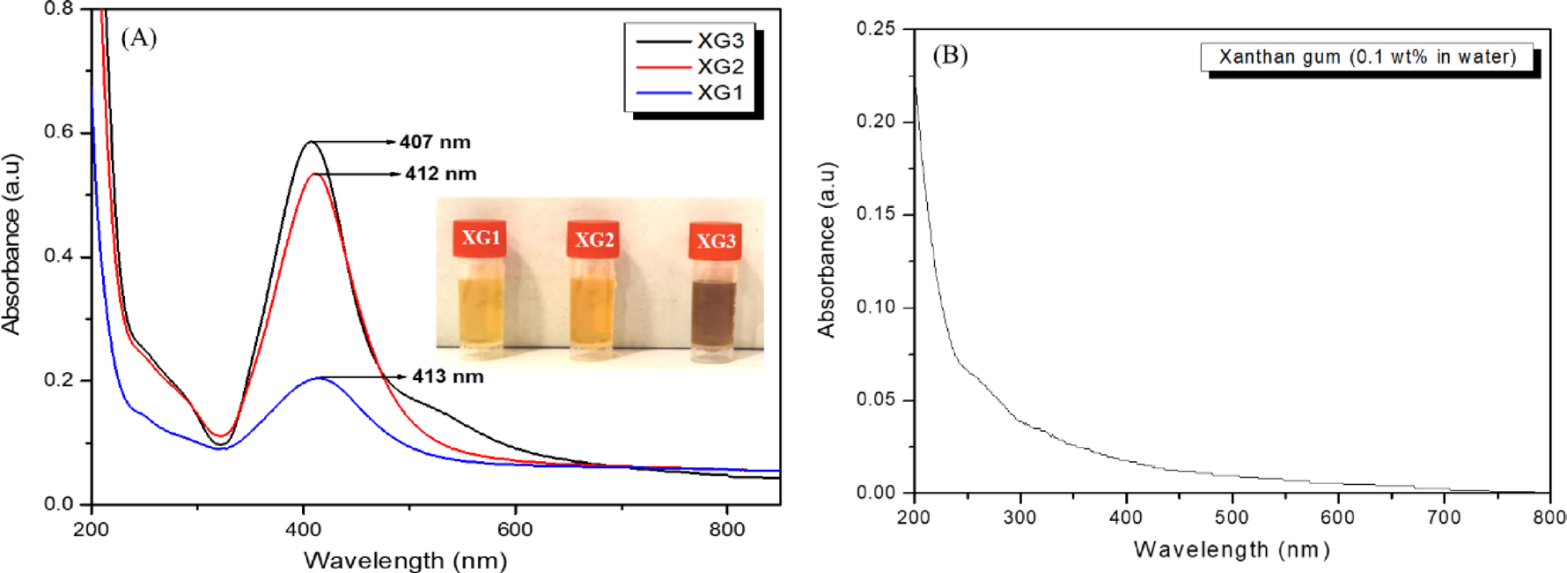
(**A**) UV-Vis absorption spectra of colloidal AgNPs (measured after 24 h). Insert: digital image of nanoparticle solutions (**B**) UV-Vis absorption of 0.1 wt% aqueous XG solution.

Green synthesis of metallic nanoparticles using plants and plant products is an environmentally friendly and economically viable process. Biopolymers such as gums, which are polysaccharides have been used to synthesize nanoparticles as these acts as mild reducing agent and a stabiliser for the nanoparticles. For a fixed XG concentration of 0.1% (w/v), the effect of increasing silver nitrate concentration on the formation of AgNPs was studied (XG1 = 0.2 mM, XG2 = 0.5 mM, and XG3 = 0.8 mM). The formation of the nanoparticles was observed visually and confirmed by UV-Vis absorption spectroscopy. A visible colour changes from colourless to pale brown and intense brown for all three samples (XG1 to XG3) indicates the formation of AgNPs, as shown in Fig. 1(A) (insert). As expected, the intensity of colour of the solution containing higher concentration of AgNPs (XG3) was high compared to solutions containing lower concentrations of nanoparticles.

The UV-Vis absorption spectra of the samples are shown in Fig. 1(A), and a well-defined surface plasmon resonance (SPR) peak is observed around 400 nm, which is characteristic of colloidal AgNPs. The SPR peak arises from the collective oscillations of free electrons at the surface conduction band when the incident light frequency matches the electrons’ natural frequency^23^. The SPR peak shows a blue shift (XG1: 413 nm, XG2: 411 nm, XG3: 407 nm), which indicates that smaller-sized nanoparticles are formed at high concentrations of silver nitrate solution. This decrease in size of the nanoparticles is primarily attributed to the availability of large number of nucleation sites for the growth of nanoparticles in the presence of XG as a template^24^. As seen in Fig. 1(A), the SPR peak’s intensity progressively increases with an increase in AgNPs in solution, which is to be expected. The shape of the SPR peak is close to symmetrical, indicating that the AgNPs synthesized using XG are spherical^25^as confirmed by the TEM images (Fig. 7). The absorption spectrum of XG did not show any absorption peak in the SPR region of AgNPs (Fig. 1(B)).

The hydroxyl groups of the polysaccharide backbone (β-1,4-linked D-glucose) of XG act as a mild reducing agent in the reduction of silver ion (Ag^+^) to AgNPs (Ag^0^). Further, the polysaccharide acts as a stabiliser for the formed AgNPs against agglomeration. Due to this two-in-one property, several kinds of polysaccharides have been used in the green synthesis of metallic nanoparticles. XG which is a polyhydroxylated biopolymer exists as a five-fold helical structure due to inter and intramolecular hydrogen bonding. This helical structure can be denatured at temperatures higher than 60 °C to expose the hydroxyl groups for the reduction process^18^.

**Fig. 2:**
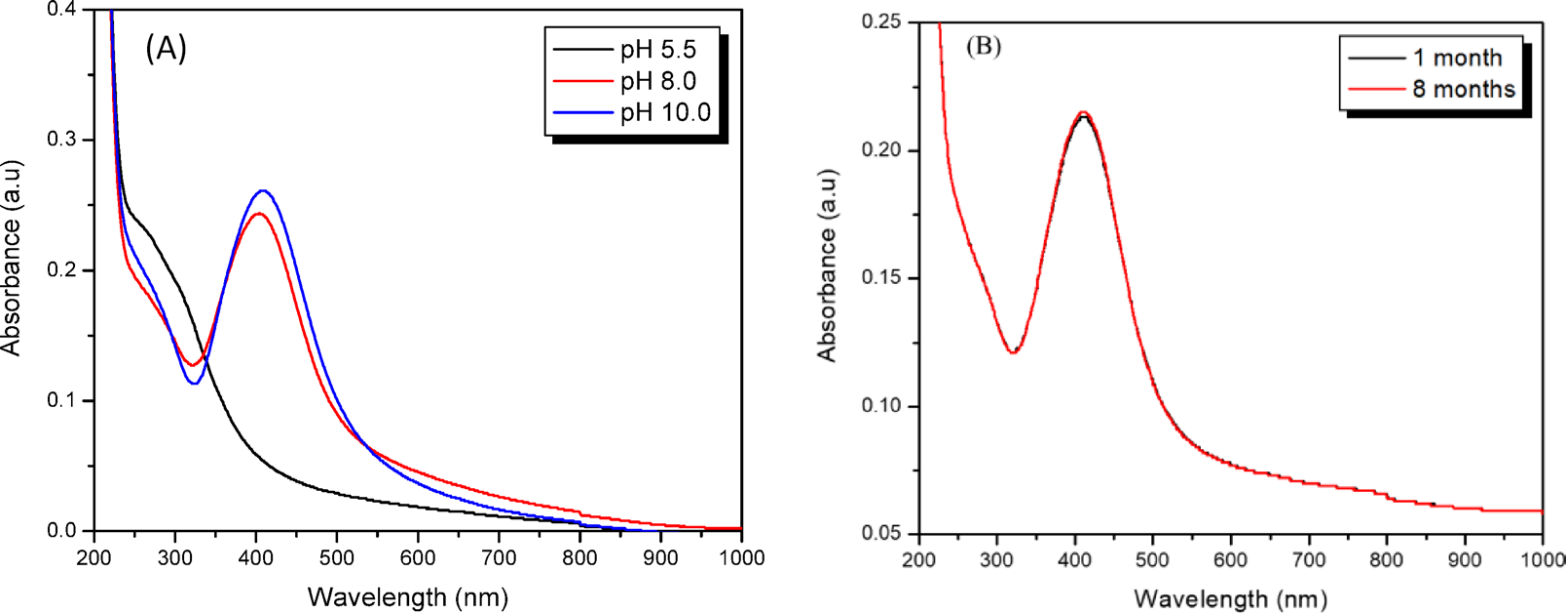
(**A**) Effect of pH on the formation of AgNPs in the presence of XG, (**B**) Stability of XG capped AgNPs after 1 month and 8 months of storage at 23 °C.

In this study, the denaturation of XG at 23 °C was achieved by changing the pH of the solution to basic condition (pH 10) by the addition of a strong base, sodium hydroxide. This denaturation exposes the many hydroxyl groups of the biopolymers which contribute to the reduction of silver ions to AgNPs and their stabilization. Primarily, at room temperature, the pH of the solution influences the reducing capability of XG. The change in pH can directly influence the deprotonation of hydroxyl groups of XG, thereby promoting the reduction of silver ions to AgNPs. The influence of solution pH on the formation of AgNPs in the presence of XG for sample XG1 is shown in Figure 2(A). The SPR peak for AgNPs is observed only for pH 8.0 and pH 10, and not at pH 5.5. At pH 5.5, the XG is in the helix form and the hydroxy groups are not fully available to act as reducing agent. However, the helix is uncoiled to random worm-like chains at pH 8.0 and pH 10 with the hydroxy groups present in the deprotonated form, which act as good reducing agents^26^. This denaturation occurs above pH 7 with complete uncoiling of the helix at pH 10. The non-covalent interaction of AgNPs with the polymer provides stability to the nanoparticles. No aggregation of the nanoparticles was observed even upon prolonged storage of the solution (8 months) at room temperature. This was confirmed by measuring the absorbance of the nanoparticle dispersion after 1 month and 8 months from the date of preparation, and the representative spectra of sample XG1 is shown in Figure 2( B). The absorbance at 413 nm was 0.213 and 0.214 after 1 month and 8 months, respectively. The insignificant change in absorbance confirms the stability of XG capped AgNPs at 23 °C.

**Fig. 3:**
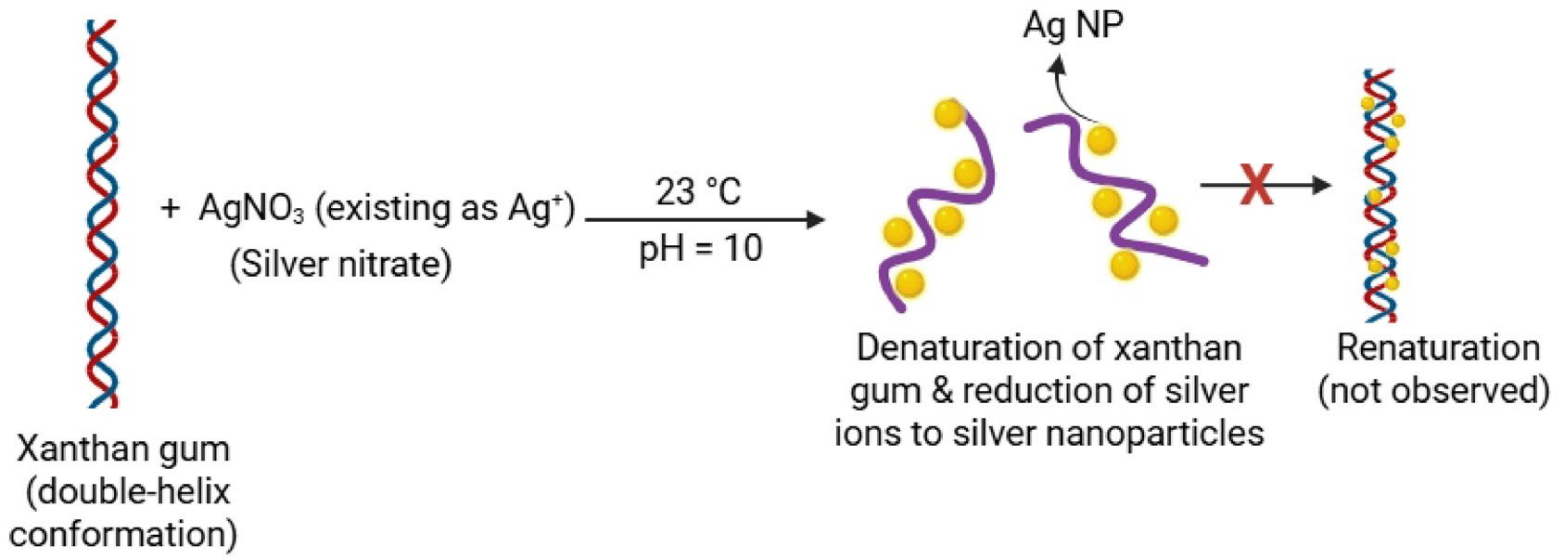
Illustration of synthesis of stable XG capped AgNPs at 23 °C and pH 10.

This further indicates that the renaturation of XG (formation of helix) in basic pH conditions did not occur at room temperature due to the steric hindrance of the nanoparticles with the chemical moieties of the polymer^26^. It has been reported that XG coated AgNPs, which were prepared at 60 °C and 100 °C, aggregated easily when stored at 4 °C for 4 weeks due to reformation of the helical and rod-like structures (renaturation)^18,27^. The scheme of synthesis and stability is illustrated in Fig. 3.

The optical bandgap of the AgNPs (XG1, XG2, XG3) was estimated by fitting the experimentally determined absorption spectra to the power law Tauc equation as^28^,

1 $$\:{\left(\alpha\:h\upsilon\:\right)}^{1/n}=A\:(h\upsilon\:-{E}_{g})$$

where *α* is the absorption coefficient, which is a function of wavelength, *h* is Planck constant, *E*_*g*_ is the optical bandgap of silver nanoparticle, *ν* is the frequency, *A* is a proportionality constant, and *n* is the Tauc exponent. The Tauc plots for the three samples are shown in.

**Fig. 4:**
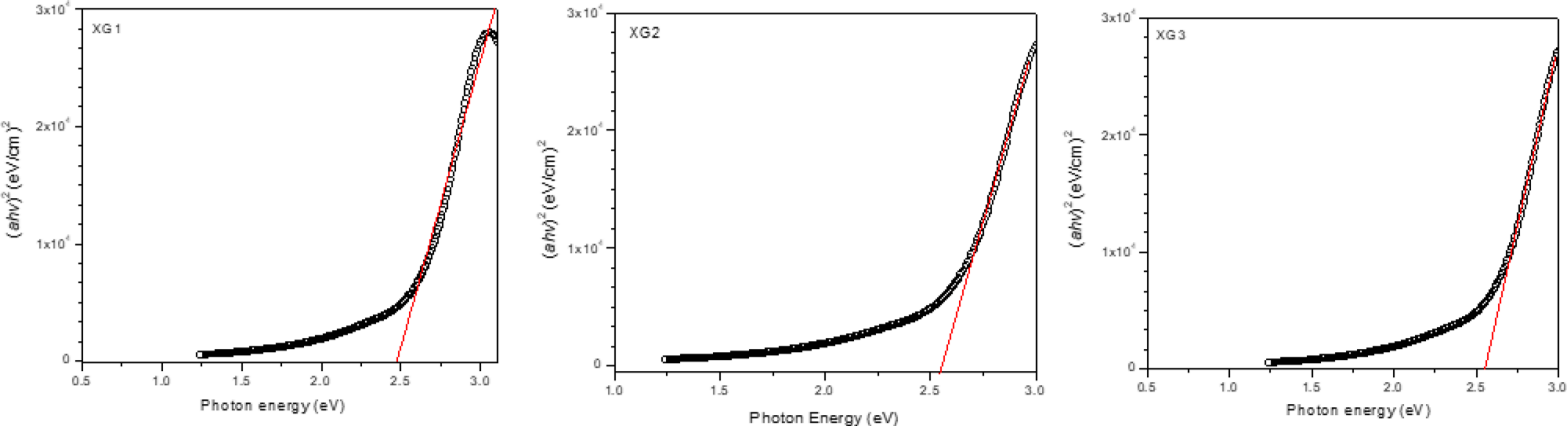
Tauc plot for colloidal XG-capped AgNPs (XG1, XG2, XG3) synthesized at 23 °C and pH 10.

Figure 4, and the optical bandgap (eV) were estimated to be 2.46 (XG1), 2.54 (XG2), and 2.55 (XG3). These values are in the range of optical band gap values that have reported for AgNPs synthesized by green approaches^26–28^.

It is also worth noting that the band gap energy increases as the size of AgNPs decreases due to quantum confinement effects. Sample XG3 with an average size of 10 nm has a bandgap of 2.55 eV, while the sample XG1 with an average size of 22 nm has a bandgap of 2.46 eV. When the size of the nanoparticles is small, the electrons and holes are confined and leads to discrete energy levels which increase the bandgap^29^.

### FTIR spectra of XG-coated AgNPs

**Fig. 5:**
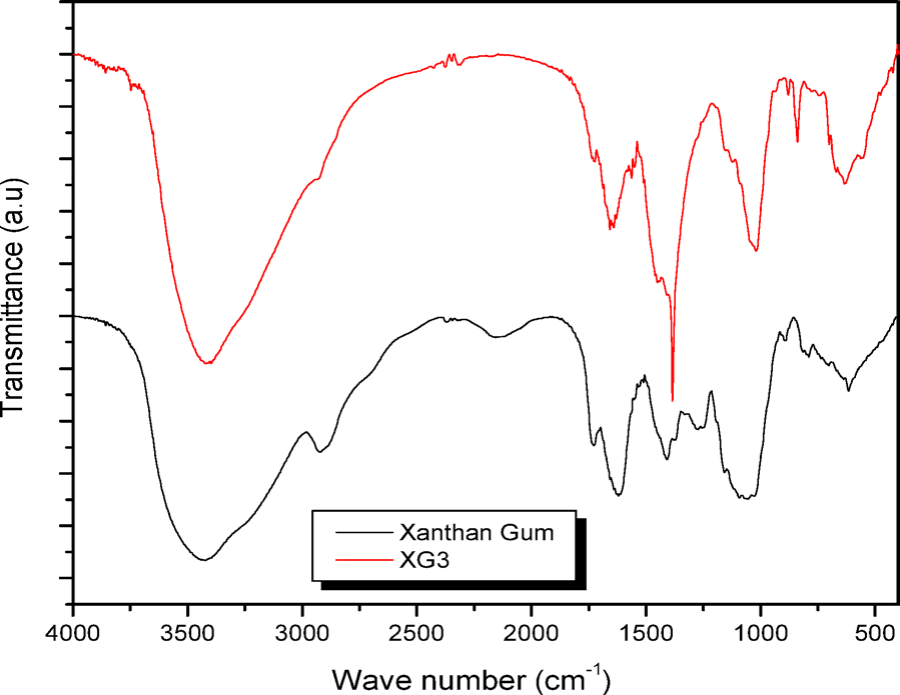
FTIR spectrum of XG and XG-capped AgNPs (XG3).

The FTIR spectra of XG and XG coated AgNPs (XG3 as a representative example) were studied to understand the capping of nanoparticles by the organic matrix. The FTIR spectra of XG and XG coated AgNPs is shown in Fig. 5. XG showed the following major characteristic absorption bands (cm^− 1^) at 3439 (broad, stretching of −OH), 2800–2950 (stretching of −CH_2_), 1732 (symmetric and asymmetric stretching of −C = O in acetyl group), 1408 (stretching of −COO), 1130 (stretching of −CO), and 919 (peak of mannopyranose). The XG capped AgNPs also exhibited major characteristic absorption bands indicating that the AgNPs are coated with organic components. The absorption peaks (-OH and -COO) are shifted to lower wavenumbers (2% in magnitude), indicating that both hydroxyl and carbonyl groups of XG are responsible for the reduction and stabilisation of AgNPs^18,30^.

### Morphology and size of AgNPs

**Fig. 6:**
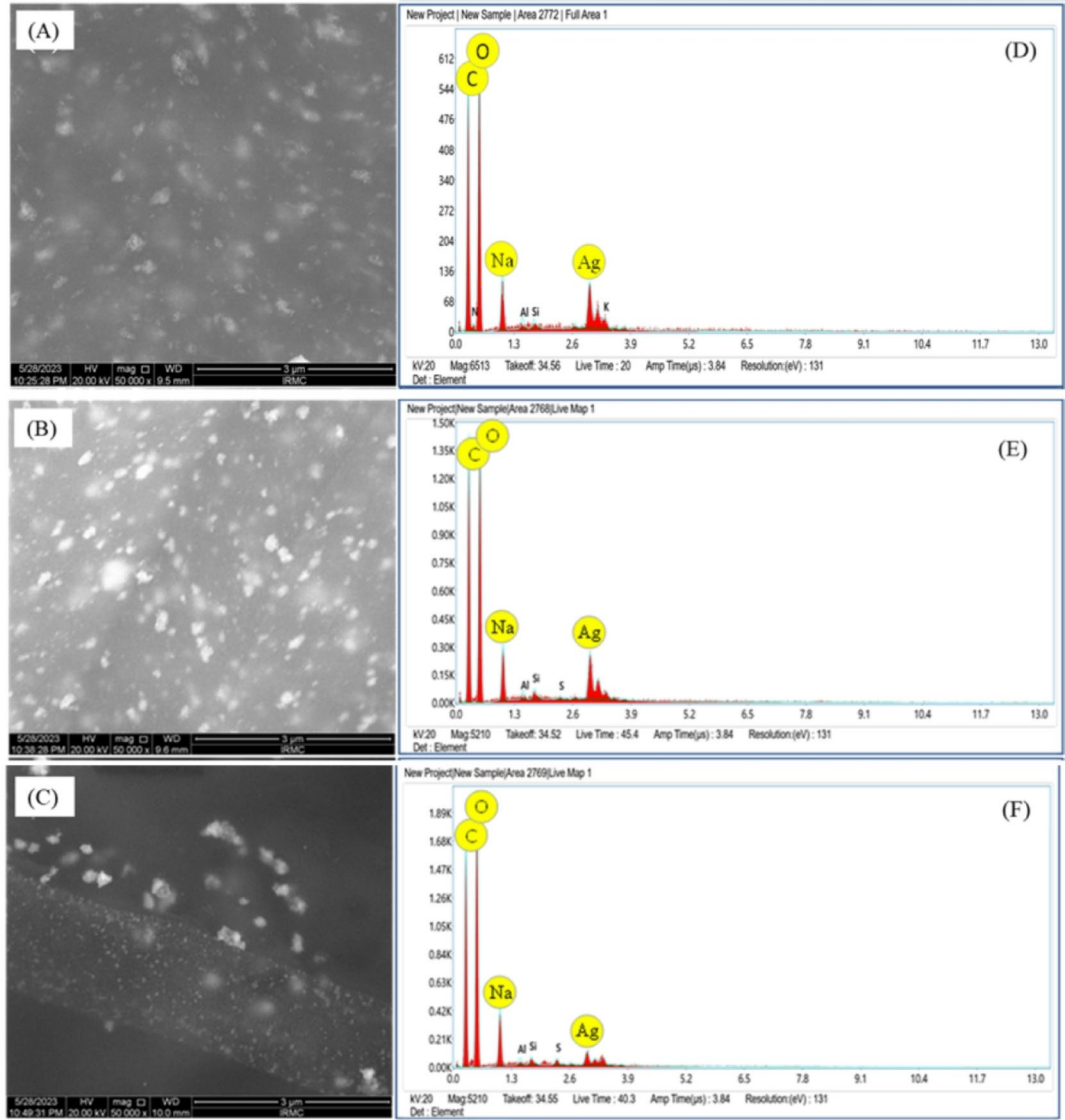
Scanning electron micrograph of AgNPs (**A**: XG1, **B**: XG2, **C**: XG3 and corresponding EDX spectrum (**D**: XG1, **E**: XG2, **F**: XG3).

**Fig. 7:**
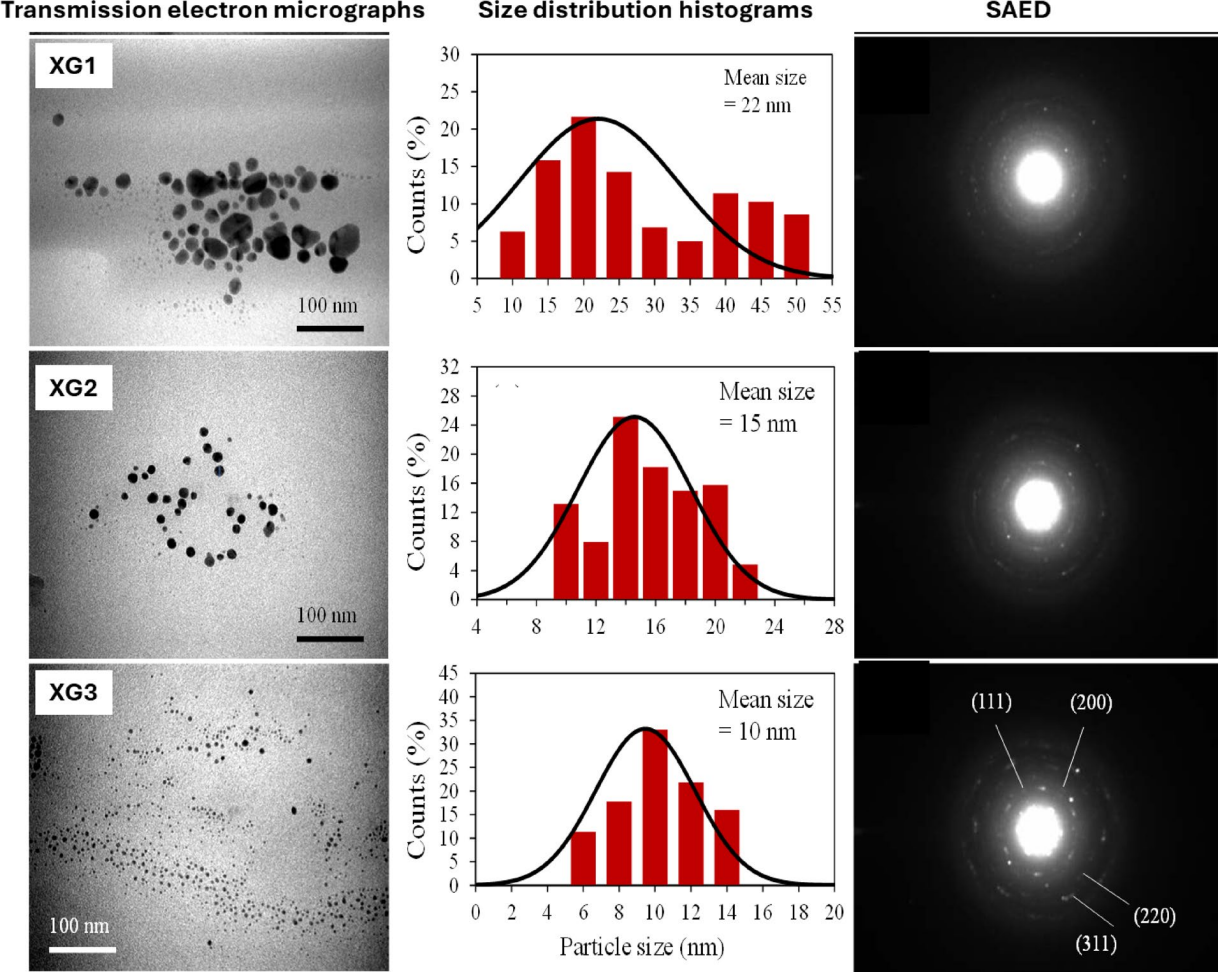
Transmission electron micrographs, size distribution histograms, and SAED pattern of XG coated AgNPs.

The morphology and size of the AgNPs were studied using electron microscopy. Clusters of close-to spherical nanoparticles capped with an organic layer (XG) is observed in the scanning electron micrographs shown in Fig. 6(A-C). The energy dispersive x-ray (EDX) spectrum corresponding to each electron micrographs shown in Fig. 6(D-F), show peaks for the various elements such as silver (Ag), carbon (C), oxygen (O), and sodium (Na) that are present in the nanoparticles. The presence of these peaks further confirms the capping of AgNPs with XG. The transmission electron micrographs, size distribution histograms, and selected area diffraction (SAED) pattern of the AgNPs are shown in Fig. 7. The nanoparticles are spherical in shape with variation in size. The average size of AgNPs were 22 nm (XG1), 15 nm (XG2), and 10 nm (XG3). The corresponding SAED pattern with characteristic Miller indices (*h*,* k*,* l* values labelled in Fig. 7) indicate that the AgNPs are crystalline in nature with face-centered cubic structure.

### Time-resolved formation of AgNPs

**Fig. 8:**
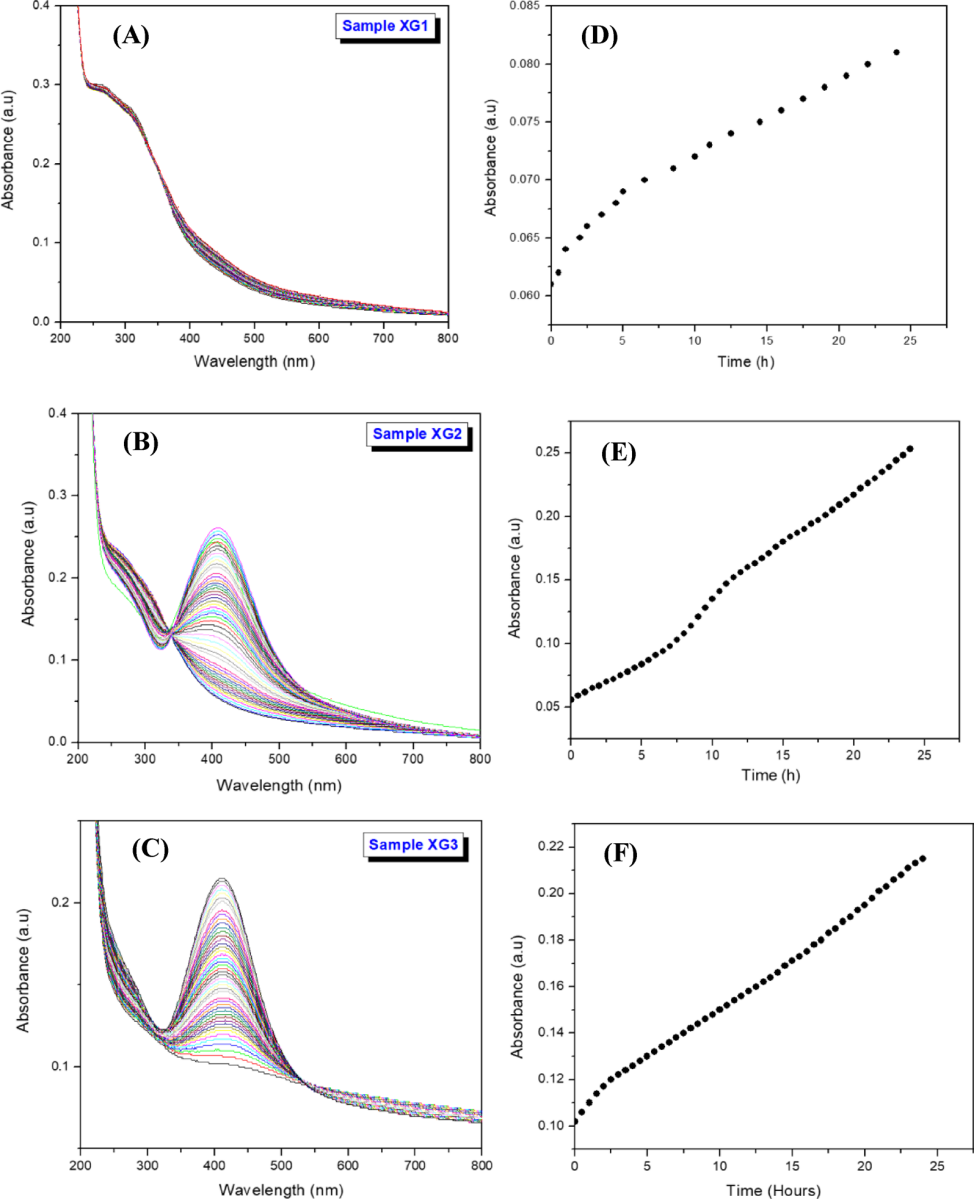
(**A**-**C**) Time-resolved absorption spectra of AgNPs formation at 23 °C and pH 10, (**D**-**F**) Change in absolute absorption maximum as function of time.

XG-mediated kinetics of formation of AgNPs was followed by measuring the absorbance of the samples as function of time at 23 °C and pH 10. The time-dependent absorption spectra of XG1, XG2 and XG3 are shown in Fig. 8 (A-C), and the corresponding absolute absorption maximum is shown in Fig. 8 (D-F). Except for XG1, the absorbance of samples XG2 and XG3 increases significantly as function of time, with the SPR peak becoming more prominent at 411 nm (XG2) and 407 nm (XG3), signifying the enhanced reduction of Ag^+^ ions to AgNPs. The effect of silver nitrate concentration on the formation of AgNPs is also evident from the absorbance measurements due to the availability of substantial number of nucleation sites at higher concentrations of AgNO_3_.

The absorption spectrum of XG1 containing 0.2 mM of AgNO_3_ shows a small shoulder at 453 nm which increases slowly over time. The initial absorbance of 0.060 reaches to a maximum of 0.080 after 24 h of reaction, due to the formation of a low concentration of AgNPs. For samples XG2 and XG3 containing 0.5 mM and 0.8 mM of Ag^+^ ions, the SPR peak increases gradually and prominently at wavelengths of 412 nm and 407 nm, respectively, indicating the enhanced formation of AgNPs. The increase in SPR peak follows a linear profile for all the three samples. A distinct isosbestic point is observed at 350 nm for XG2.

XG is a polysaccharide with helical structure that can denature to a random coil at temperatures higher than 60 °C. Under the experimental conditions, the helical structure denatures, exposing the active hydroxyl groups on the polymer backbone relatively slowly, leading to the reduction of Ag^+^ ions to Ag^0^ nanoparticles. The formed nanoparticles become embedded in the polymer coil and are stabilised against aggregation. It has been reported that the reduction can be enhanced by heating the solution to 100 °C for 24 h. This allows for the hydrogen bonds to be weakened and to expose the hydroxyl groups that are involved in the reduction process^18^. The temperature-dependent kinetics of denaturation and renaturation of XG has been confirmed through rheology, circular dichroism, and small-angle x-ray scattering (SAXS) studies^31^.

**Fig. 9:**
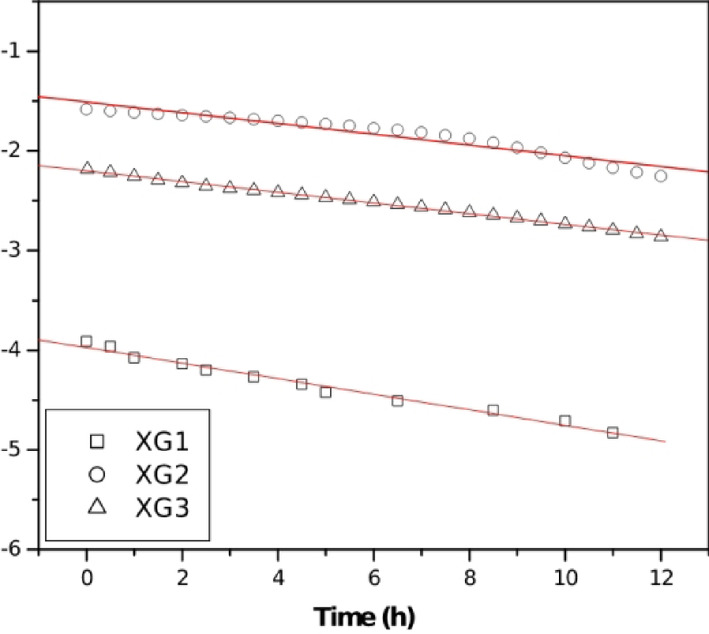
First-order kinetics plot for the formation of AgNPs at 23 °C and pH 10. (Red line is the linear regression fit).

The formation kinetics of AgNPs was evaluated by fitting the experimental absorption data (up to 12 h) to the first-order kinetic model as^32^,

2 $$\:\text{ln}\left({A}_{inf}-{A}_{t}\right)=\:-kt$$

where *A*_inf_, and *A*_*t*_ are the absorbance at infinite time (after 24 h), and at time *t*, respectively, *k* is the rate constant, and *t* is the time in hours. The first order rate constant was obtained from the slope of a plot of ln(*A*_∞_-*A*_0_) versus *t*, (Fig. 9) and the results are summarized in Table 1.

**Table 1:** Reaction rate constants (*k*) and linear regression coefficients (*r*^2^ for the formation of AgNPs at 23 °C and pH 10.

Sample	[AgNO_3_]	Size (nm)	First-order kinetic model	
	(mM)		k (h^− 1^)	*r* ^2^
XG1	0.2	22 ± 0.5 nm	0.053	0.996
XG2	0.5	15 ± 0.5 nm	0.055	0.987
XG3	0.8	10 ± 1.0 nm	0.079	0.998

The rate constant, *k*, is in the range (0.053 h^− 1^ to 0.079 h^− 1^) and increases with an increase in the concentration of silver nitrate in the solution at 23 °C. The rate constants are significantly smaller than those reported for AgNPs synthesized at 60 °C ( *k* = 0.0026 s^− 1^) and 100 °C (*k* = 0.0109 s^− 1^). This large difference in rate constants explains the influence of temperature, pH and concentration in the helix-to-random coil transformation of XG. At high temperatures, the hydroxyl groups are exposed and readily involved in the reduction of silver nitrate and stabilisation of the nanoparticles^30^. It is also observed that the rate constant increases with decease in the size of the nanoparticles. As the size of the nanoparticles becomes smaller, the surface area increases, thereby increasing the reaction rate. Similar observations have been reported for silver-coated magnetic nanoparticles^33^.

### Degradation of 2-nitrophenol

**Fig. 10:**
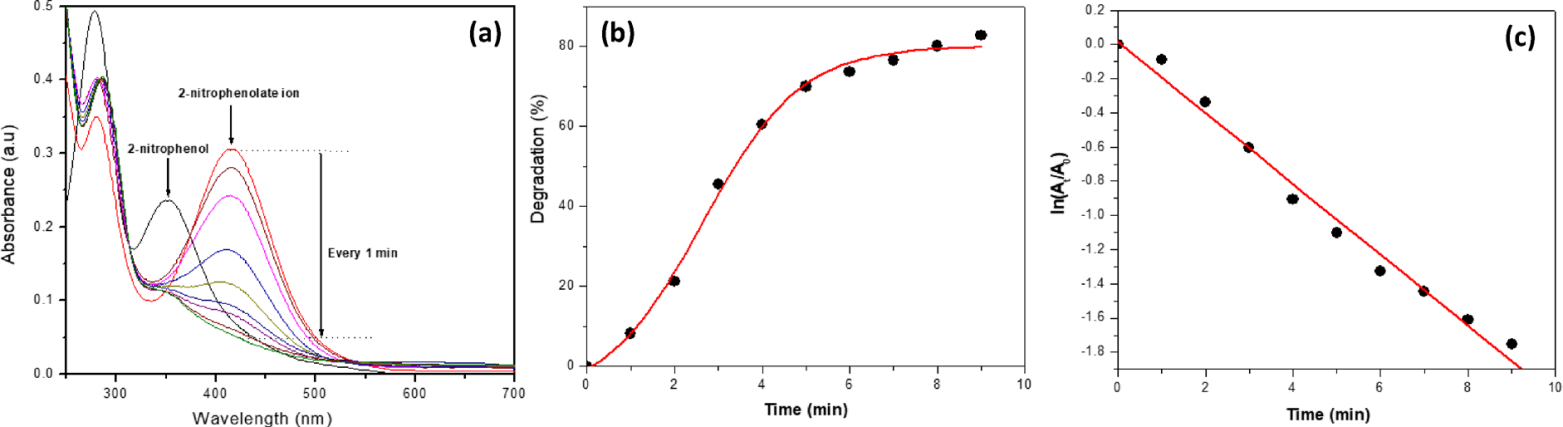
Catalytic reduction of 2-nitrophenol with XG capped AgNPs (XG3) at 23 °C.

The catalytic potential of the AgNPs (XG3) was studied using 2-nitrophenol as a model organic pollutant. The sample XG3 was chosen for this study due to its small size and ease of formation. The 2-nitrophenol pollutant is an environmental hazard and, above the threshold concentration, causes methemoglobinemia in humans. This compound upon reduction leads to the formation of 2-amino phenol, which is non-toxic. The time-dependent change in absorbance that corresponds to the conversion of 2-nitrophenol to 2-aminophenol in the presence of NaBH_4_ is shown in Fig. 10 (A). The absorption peak at 352 nm which corresponds to 2-nitrophenol is shifted to 416 nm upon adding NaBH_4_, due to the formation of a 2-nitrophenolate ion. This ion results from deprotonation of the -OH group of the phenolic group of 2-nitrophenol. The change (decrease) in absorption peak intensity at 417 nm as function of time corresponds to the formation of 2-aminophenol.

However, this reaction has a large kinetic barrier due to the high potential difference between the reactant (2-nitrophenol) and the reducing agent (NaBH_4_)^34^thereby requiring a catalyst to overcome the large energy barrier associated with the reduction process at room temperature. With the addition of AgNPs (XG3), a significant decrease in the intensity of absorption peak at 416 at room temperature (23 °C) is clearly observed due to the reduction of 2-nitrophenol to 2-aminophenol. The initial absorbance of 0.306 at 417 nm reduces to 0.05 within the first 9 min of reaction, and this amounts to about 80% degradation of 2-nitrophenol. This fast reaction is brought about by the catalytic properties of XG coated AgNPs. The percentage degradation was calculated using the expression,

3 $$\:\text{Degradation}\:\left(\text{\%}\right)=\frac{{A}_{0}-{A}_{t}}{{A}_{0}}\times\:100$$

where *A*_0_ and *A*_t_ are the initial absorbance and absorbance at time *t*, respectively.

(**A**) Change in absorbance spectra as function of time, (**B**) Plot of degradation (%) as function of time, (**C**) Pseudo-first order kinetic plot.

The plot of percentage degradation as function of time is shown in Fig. 10 (B). The degradation follows a sigmoidal pattern within the limits of experimental error reaching about 80% in just 9 min. The degradation reaction follows a pseudo-first order reaction kinetics as the concetration of NaBH_4_ was much higher (10 mM) compared to 2-nitrophenol (1 mM). The pseudo-first order rate constant was determined to be 0.211 min^− 1^ from the gradient of a plot of ln (*A*_*t*_/*A*_0_) versus time as shown in Fig. 10 (C), according to the following equation,

4 $$\:\text{l}\text{n}\frac{{C}_{t}}{{C}_{0}}=ln\frac{{A}_{t}}{{A}_{0}}=-kt$$

where *C*_*t*_ and *C*_0_ are the concentration of 2-NP, and *A*_*t*_ and *A*_0_ are the absorbances at time *t*, and *t* = 0, respectively, *k* (min^− 1^) is the rate constant of the reaction.

**Fig. 11:**
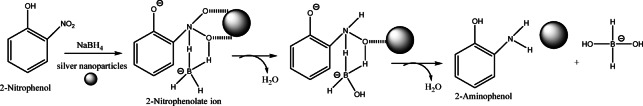
Mechanism of catalytic degradation of 2-nitrophenol in the presence of AgNPs and NaBH_4_ at 23 °C.

The reaction kinetics and the rate constant value are in close agreement with reported values for catalytic reduction of 2-nitrophenol and 4-nitrophenol using green synthesized nanoparticles^18,35–37^. However, the rate constant 0.211 min^− 1^ determined in this work for the degradation of 2-nitrophenol is much higher than the rate constant 0.133 min^− 1^, reported for the degradation of hexacyanoferrate (II) by XG capped AgNPs^19^. In general, differences in rate constants arises due to difference in the size of nanoparticles and method of synthesis. Based on these observations, a schematic representation of the catalytic degradation of 2-nitrophenol by XG-capped AgNPs is illustrated in Fig. 11.

The reduction process generally follows the Langmuir-Hinshelwood kinetic model with the following mechanism^38^. The reactant molecules (2-nitrophenol) first move to the surface of AgNPs with reversible adsorption. The catalytic reduction then occurs on the surface of the nanoparticles, and the rate is dependent on the amount of reactant adsorbed. The electron transport between the reactant and NaBH_4_ at the active site of the nanoparticles allows the formation of the end product, 2-aminophenol. Desorption of the product from the surface of the catalyst takes place with an increase in concentration of the product, thereby providing vacant sites on the catalyst for further reaction.

#### Antibacterial activity

**Fig. 12:**
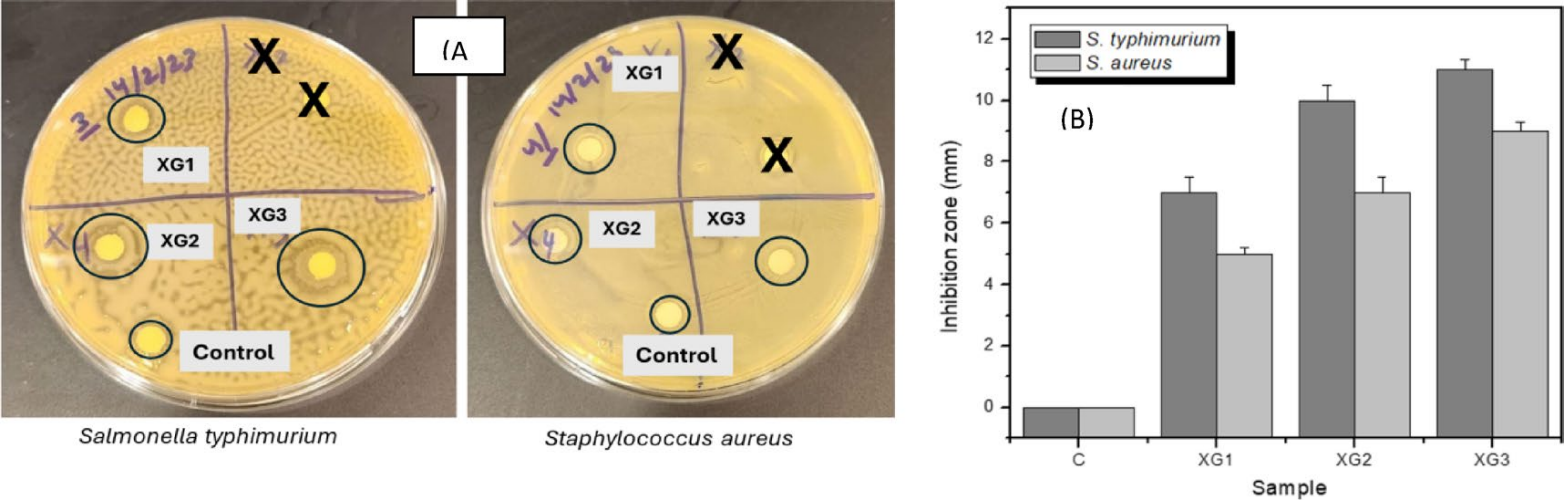
(**A**) Digital images of bacterial culture plates showing zone of inhibition, (**B**) Plot of zone of inhibition for XG decorated AgNPs against *S. typhimurium*, and *S. aureus*.

The antibacterial potential of the XG decorated AgNPs was evaluated against *S. typhimurium* and *S. aureus*, by disc diffusion method. The clear inhibitory zone observed around the discs containing the sample after incubation (24 h) indicate the antibacterial activity of the nanoparticles against the two types of bacteria (Fig. 12A). The measured zone of inhibition of bacterial for the three nanoparticle samples is shown in Fig. 12B.

For the same dosage of nanoparticles, the zone of inhibition is more prominent for *S. typhimurium* than for *S. aureus* which is attributed to different levels of activity of the nanoparticles on the bacterial cell wall. The antibacterial activity arises due interaction of the nanoparticles with bacterial cell leading to multiple toxic effects such as lipid peroxidation, formation of reactive oxygen species, protein oxidation and DNA degradation. The interactions are mainly ionic in nature between the negatively charged bacterial cells and positively charged nanoparticles^39,40^. The effect of the size of AgNPs on the antibacterial is also evident in Fig. 12(B). The small nanoparticles (sample XG3, size 10 nm) show a larger effect than the large nanoparticles (sample XG1, size 22 nm). ANOVA analysis revealed a significant effect of nanoparticle size on bacterial growth inhibition with a P-value of 0.010. The small nanoparticles are more effective in penetrating the bacterial cell than the large ones, causing higher bacterial inhibition due to significant generation of reactive oxygen species and membrane damage^41,42^.

## Conclusion

Stable AgNPs in the 10–22 nm size were successfully synthesised using a polysaccharide biopolymer, XG, as a reducing and stabilising agent. The denaturation of XG from a helix to a worm-like polymer (random coil) was observed at pH 10, exposing the hydroxyl groups responsible for the reduction process. No aggregation due to renaturation was observed in this study, even after prolonged storage of the material at room temperature room temperature. The presence of XG capping on the surface of the nanoparticles was confirmed using FTIR spectroscopy. The nanoparticles exhibited good antibacterial and catalytic properties. Using natural biopolymers (gums) in the green synthesis of nanoparticles can be exploited for size and morphology control for various targeted biomedical applications.

## Methods

### Materials

XG, silver nitrate, sodium hydroxide, sodium borohydride, and 2-nitrophenol were purchased from Sigma-Aldrich and used as received. All aqueous solutions were prepared using deionized water with an electrical conductivity of 18.2 MΩ cm^−1^, obtained from a Milli-Q water purification system (Elix Technology, Germany). The antibacterial properties of the synthesized AgNPs were evaluated against two different types of bacteria, *Escherichia coli* (*E. coli*) and *Salmonella typhimurium* (*S. typhimurium*). The bacteria were obtained from the Ministry of Health (MOH, Bahrain).

### Preparation of XG aqueous solution (XG)

A 0.1% (w/v) aqueous solution of XG was prepared by dissolving 0.10 g of XG in 100 ml of water at 75 °C for complete solubility. The solution was centrifuged at 4000 rpm for 10 min to remove any suspended particles. The supernatant was transferred to a clean glass vial and stored in the refrigerator.

### Synthesis of AgNPs

The AgNPs were prepared by mixing known amounts of silver nitrate solution and XG solution at room temperature. Three sets of samples (XG1 to XG3) were prepared, and the compositions are listed in Table 2. The preparation of sample XG1 is described as a representative example as follows. XG solution (0.1% w/v, 2 ml) was added dropwise to silver nitrate solution (0.4 mM, 8 ml) under gentle magnetic stirring at 23 °C. The mixture was stirred for 10 min, followed by the addition of sodium hydroxide solution (1 M, 50 µl). Upon adding the sodium hydroxide solution, the reaction mixture turned from colourless to pale yellow indicating the formation of colloidal AgNPs. The pH of the mixture was maintained at 10. The reaction mixture was stirred for another 24 h to complete the formation of AgNPs. After 24 h, the colour of the mixture turned more intense, indicating complete reduction and formation of nanoparticles. The nanoparticles were isolated by repeated centrifugation (13,000 rpm) and washing with water. The washed nanoparticles were lyophilised and stored in a desiccator until further use.

**Table 2:** Sample composition for XG mediated synthesis of AgNPs at 23 °C and pH 10.

Sample^†^	XG solution (0.1% w/v) (ml)	AgNO_3_ [Initial] (ml)	AgNO_3_ [Final]	Colour after 24 h
XG1	5	0.4 mM (5)	0.2 mM	Pale yellow
XG2	5	1.0 mM (5)	0.5 mM	Pale yellow
XG3	5	1.6 mM (5)	0.8 mM	Light brown

^†^all samples contain 50 µl of 1 M sodium hydroxide, pH 10.

### Characterization

#### UV-Vis absorption spectroscopy

The formation of AgNPs was confirmed by measuring the absorption spectrum of the samples using a double-beam Shimadzu UV-1800 spectrophotometer. The sample (3 ml) was placed in a quartz cuvette of 1 cm path length (Helma Analytics, Germany), and the absorption spectrum was recorded in the wavelength range 350–800 nm with a spatial resolution of 1 mm.

### Scanning electron microscopy (SEM) and Energy-Dispersive X-ray spectroscopy (EDX)

The chemical compositions of the AgNPs were determined using a scanning electron microscope equipped with an energy-dispersive X-ray (EDX) detector (TESCAN, VEGA 3, 20 kV, Tescan Orsay Holding, Brno, Czech Republic).

### Transmission electron microscopy (TEM)

The AgNPs were characterized using FEI Morgagni 268 transmission electron microscope (TEM) operating at an accelerating voltage of 80 kV. The samples were prepared by depositing a drop of the colloidal nanoparticles on a carbon-support copper grid. The grids were air-dried and transferred to the microscope. The AgNPs were imaged in the bright-field mode.

### Fourier transform infrared (FTIR) spectroscopy

The infrared spectra of XG, and XG coated AgNPs, were recorded using a Bruker Alpha spectrophotometer (Bruker Inc., Madison, WI, USA) in the scanning range of 500–4000 cm^−1^. The samples were ground with dry potassium bromide (spectroscopic grade, Sigma-Aldrich) into fine powder using an agate mortar and pestle. The powder was pressed into a thin transparent pellet using a pellet-making machine.

### Catalytic study

The catalytic potential of XG stabilised nanoparticles (XG3) was studied using 2-nitrophenol as a model organic pollutant in the presence of a sodium borohydride (NaBH_4_) at 23 °C. A 2.5 ml of a 0.1 mM 2-nitrophenol solution was placed into a quartz cuvette and the absorption spectrum recorded on a Shimadzu UV-Vis spectrophotometer (UV-1800) in the wavelength range of 200–700 nm. A 0.5 ml of NaBH_4_ (0.1 M) was added to this solution, and the absorption spectrum was recorded. Following this, the nanoparticle (XG3) sample (100 µl containing 0.00040 g of silver nanoparticle) was added, and the absorption spectra were recorded for every 1 min.

### Antibacterial studies

The antibacterial properties of the synthesized AgNPs against a Gram-positive, *Staphylococcus aureus* (*S. aureus*), and a Gram-negative, *Salmonella typhimurium* (*S. typhimurium*). bacteria were carried out using the Kirby-Bauer disk diffusion susceptibility test method. The bacteria strains were spread in all directions on a nutrient agar (LB agar) medium using a sterile spreader with growth allowed for 24 h. With aseptic precautions, the filter paper (Whatman) disks were loaded with the nanoparticles and placed on the agar plate. The plates were incubated for 24 h at 37 °C, to determine the zone of inhibition. All measurements were conducted in triplicate and the average values of inhibition zones were calculated as Mean ± Standard deviations. The data was statistically analysed using Origin software.

## Data Availability

The datasets used and/or analysed during the current study are available from the corresponding author on reasonable request.
